# How do pediatric patients perceive adverse drug events of anticonvulsant drugs? A survey

**DOI:** 10.1007/s00431-020-03571-1

**Published:** 2020-03-11

**Authors:** Martina Patrizia Neininger, Sarah Woltermann, Sarah Jeschke, Birthe Herziger, Ruth Melinda Müller, Wieland Kiess, Thilo Bertsche, Astrid Bertsche

**Affiliations:** 1 Drug Safety Center and Clinical Pharmacy, Institute of Pharmacy, Medical Faculty, Leipzig University, Brüderstraße 32, 04103 Leipzig, Germany; 2 Center for Pediatric Research, University Hospital for Children and Adolescents, Liebigstraße 20a, 04103 Leipzig, Germany; 3 Neuropaediatrics, University Hospital for Children and Adolescents, Ernst-Heydemann-Str. 8, 18057 Rostock, Germany

**Keywords:** Adverse drug events (ADEs), Anticonvulsants, Children, Adolescents, Perceptions, Interview

## Abstract

Anticonvulsant drugs have a high risk of adverse drug events. Little is known about the perception of those events by pediatric patients. We performed a survey in the neuropediatric departments of two university hospitals. Using a questionnaire, we interviewed patients aged 6–18 years with current anticonvulsant treatment regarding (i) their fears about potential adverse drug events, (ii) experienced adverse drug events, and (iii) perceived burden of experienced adverse drug events. One hundred patients took part in the interview. (i) 40 (40%) expressed fears that the medication could harm them. Eighteen of 40 (45%) named fears concerning specific adverse drug events. Of those, 12/18 (67%) feared neurologic or psychiatric symptoms. (ii) 37 (37%) of children described altogether 60 experienced adverse drug events. Of those, 38 (63%) concerned neurologic or psychiatric symptoms. (iii) 32/37 (82%) children who experienced adverse drug events felt bothered by the experienced event. Among others, they described an emotional burden (11/37, 30%), and restrictions in school performance (8/37, 22%) and favorite leisure activities (4/37, 11%).

*Conclusion*: School-aged children are well able to describe adverse drug events of their anticonvulsant medication. Almost two thirds of the described events concern neurologic or psychiatric symptoms that cause an emotional burden and restrictions according to the patients.

**Table:** 

**What is Known:** *• Anticonvulsants have a high potential of adverse drug events.* *• In an earlier survey, parents expressed fears of severe adverse drug events such as liver failure, which seldom occur, and reported a high number of neurological and psychological adverse drug events.*	
**What is New:** *• Many children fear that their anticonvulsants could harm them, and they fear and experience neurological and psychological adverse drug events.* *• According to the children, adverse drug events cause an emotional burden and restrictions in school performance and favorite leisure activities.*

**What is Known:**

*• Anticonvulsants have a high potential of adverse drug events.*

*• In an earlier survey, parents expressed fears of severe adverse drug events such as liver failure, which seldom occur, and reported a high number of neurological and psychological adverse drug events.*

**What is New:**

*• Many children fear that their anticonvulsants could harm them, and they fear and experience neurological and psychological adverse drug events.*

*• According to the children, adverse drug events cause an emotional burden and restrictions in school performance and favorite leisure activities.*

## Introduction

Anticonvulsant drugs have a high potential of adverse drug events (ADEs) [4, 20]. Information on those ADEs is usually derived from randomized controlled trials conducted for drug approval of the drugs. Data from routine care, however, are scarce. In the available studies, especially psychiatric and behavioral ADEs of anticonvulsant drugs were observed in adult patients [5, 18, 25]. Earlier surveys on routine anticonvulsant medication in children have shown that perception of ADEs can lead to discontinuation of anticonvulsant therapy [3] and to reduction of quality of life [16]. Besides, the chance that parents turn to complementary and alternative medicine for their child’s epilepsy has been shown to be 5.6-fold higher if they perceive ADE of the child’s anticonvulsant medication [14]. For this reason, earlier, a parents’ survey on their perception of adverse drug events was performed. Parents feared especially serious ADEs such as liver failure that fortunately seldom occur. However, they described a high number of experienced ADEs, especially sedation and abnormal behavior. They reported that those ADEs have a substantial negative impact on their children’s daily life and development [2]. Although it has been shown that pediatric epilepsy patients are well able to report implications of their epilepsy [22] and many children and adolescents have at least some knowledge on their medication [10, 22], it remains unclear if the perception of ADEs of anticonvulsants by the pediatric patients themselves matches the parents’ concerns and experiences. Thus, we performed a survey of children and adolescents suffering from epilepsy on their fears and experiences concerning ADEs of their anticonvulsant medication, the burden of experienced ADEs, and measures taken in case of an ADE.

## Materials and methods

### Setting and patients

After approval of the responsible ethics committees, we performed this prospective observational study in the neuropediatric departments of two university hospitals in Germany for 6 months. We consecutively invited patients aged 6–18 years with a diagnosed epilepsy and their parents to participate in the interview. We approached the patients and their parents during a hospital stay or appointment in the outpatient clinic. As the study was targeted at ADEs of anticonvulsant long-term treatment, we included only patients receiving at least one drug as anticonvulsant long-term medication. Patients who were only prescribed rescue medication were excluded. Patients whose accompanying persons were not their legal guardians were not included in the study. Furthermore, children unable to understand and answer the questions were excluded. Written informed consent was obtained from all participating children and their parents. Participation was voluntary.

### Data assessment

An expert panel consisting of child neurologists, pharmacists, and a pedagogue in advanced postgraduate training in child and adolescent psychotherapy developed a questionnaire to facilitate a structured and standardized interview. The questionnaire consisted of open questions to grasp the bandwidth of the children’s perceptions and answers. In the data evaluation, the children’s answers were clustered by the expert panel. We asked the following questions:

1. Do you fear that your medication could harm you or your body? If yes, which fears do you have?
2. Have you already experienced a side effect? If yes, which one(s)?
3. What did bother you the most about the experienced side effect?
4. What did you and your parents do in case of a side effect?

Further, we collected data on current anticonvulsant medication prescription, epilepsy diagnosis, and sociodemographic data from the medical records. Besides, we gained information from the medical records on how physicians reacted to the ADEs described by the pediatric patients.

### Statistics

We report frequencies in absolute and relative numbers. Continuous data are presented with median, quartiles, and minimum/maximum. We applied a chi-square test to compare the number of mono and combination therapies in which an ADE occurred. A *p* value ≤ 0.05 was considered to indicate significance.

## Results

We interviewed a total of 100 children. Patients’ characteristics and information on the children’s epilepsy and medications are displayed in Table 1.

**Table 1:** Characteristics of patients and their current medications

Characteristic	Patients
Total number (*n*) (male/female)	100 (54/46)
Median age (Q25/Q75; min./max.) (years)	11.7 (8.7/14.7; 6.3/17.3)
Type of epilepsy (*n* (%))	Rolandic epilepsy 21 (21%)
	Other focal epilepsy 39 (39%)
	Absence epilepsy 19 (19%)
	Other generalized epilepsy 14 (14%)
	Unspecified epilepsy 7 (7%)
Median duration of epilepsy (Q25/Q75; min./max.) (years)	2.7 (0.9/5.4; 0.0/15.1)
Current anticonvulsant therapy regimen	
Total number of anticonvulsant drugs (*n* (% of patients))	131
Levetiracetam	33 (33%)
Sulthiame	26 (26%)
Lamotrigine	20 (20%)
Ethosuximide	16 (16%)
Oxcarbazepine	13 (13%)
Valproate	11 (11%)
Lacosamide	6 (6%)
Topiramate	3 (3%)
Clobazam	1 (1%)
Zonisamide	1 (1%)
Primidone	1 (1%)
Thereof monotherapies (*n* (% of patients))	74 (74%)
Combined medication schemes (*n* (% of patients))	26 (26%)
Combination of 2 drugs	21 (21%)
Combination of 3 drugs	5 (5%)

### Fears concerning the medication

Of all 100 children, 40 (40%) reported that they fear that the medication could harm them or their body. Those 40 children expressed fears concerning (multiple answers possible) specific ADEs (18/40; 45%), diffuse fears (8/40, 20%), loss of effectiveness (6/40; 15%), negative impact on daily life and/or school/sports performance (5/40; 13%), death (3/40; 8%), and others (5/40; 13%). Those 18 children who feared specific ADEs named altogether 22 specific ADEs, most frequently fatigue (5/18; 28%). Of the feared specific ADEs, 13/22 (59%) concerned neurologic or psychiatric symptoms expressed by 12/18 (67%) children (Table 2).

**Table 2:** Feared and experienced adverse drug events (ADEs); the table is sorted according to the frequency of experienced ADEs in the categories “neurologic and psychiatric” and “non-psychiatric/non-neurologic”

Adverse drug event (ADE)	Feared ADE *n*	Feared ADE % (/18 who expressed fears of specific ADE)	Feared ADE % (/100 participants)	Experienced ADE *n*	Experienced ADE % (/37 who expressed experience of ADE)	Experienced ADE % (/100 participants)
Neurologic and psychiatric						
Fatigue	5	28	5	11	30	11
Disturbance in attention	2	11	2	6	16	6
Headache	1	6	1	5	11*	4*
Mood swings	1	6	1	3	8	3
Aggression	0	0	0	2	5	2
Depressive symptoms	1	6	1	2	5	2
Insomnia	1	6	1	2	5	2
Unsteady gait	0	0	0	2	5	2
Vertigo/dizziness	0	0	0	2	5	2
Agitation	0	0	0	1	3	1
Mydriasis	0	0	0	1	3	1
Tremor	0	0	0	1	3	1
Absences/increased number of seizures	2	12	2	0	0	0
Non-psychiatric/non-neurologic						
Increased appetite/weight gain	1	6	1	5	14	5
Vomiting	1	6	1	5	14	5
Nausea	2	11	2	3	8	3
Dyspnea	1	6	1	2	5	2
Decreased appetite/weight loss	1	6	1	2	5	2
Diarrhea	0	0	0	1	3	1
Exanthema	1	6		1	3	1
Fever	0	0	0	1	3	1
Liver injury	2	11	2	1	3	1
Stomach ache	0	0	0	1	3	1

*One patient described headache caused by two different monotherapies

### Experienced ADEs

Of all 100 children, 37 (37%) reported to have experienced at least one ADE: 22/37 (59%) named one ADE, 9/37 (24%) 2 ADEs, 4/37 (11%) 3 ADEs, and 2/37 (5%) 4 ADEs. Of the patients suffering from rolandic epilepsy 7/21 (33%), of those with other focal epilepsies 15/39 (38%), of those with absence epilepsies 9/19 (47%), of those with other generalized epilepsies 3/14 (21%), and of those with unspecified epilepsies 3/7 (43%) described one or more experienced ADEs. Altogether, 60 ADEs were reported by those 37 children describing ADEs, most frequently fatigue (11/37; 30%) and disturbance in attention (6/37; 16%, Table 2). Of the reported experienced ADEs, 38/60 (63%) concerned neurologic or psychiatric symptoms. In 7/60 (12%) ADEs, the patients assigned the ADEs to a specific anticonvulsant drug. In another 48/60 (80%), the ADE was assigned to an anticonvulsant drug according to the information from the patients’ medical records (Table 3). In 49/60 (82%), the ADE occurred during monotherapy, in 10/60 (17%) during combination therapy, and in 1/60 (2%) the therapy mode at the time of the occurrence of the ADE was unclear. ADEs as described by the pediatric patients occurred in 34/81 (42%) monotherapies and 6/22 (27%) combination therapies (n.s.). In 46/60 (77%), the ADEs occurred during long-term therapy, in 8/60 (13%) at the initiation of the therapy, and in 6/60 (10%) the time of occurrence could not be determined from the patients’ medical records.

**Table 3:** Experienced adverse drug events (ADEs) assigned to the anticonvulsant drug hold responsible; *ESM* ethosuximide, *LTG* lamotrigine, *LEV* levetiracetam, *OXC* oxcarbacepine, *STM* sulthiame, *TPM* topiramate, *VPA* valproic acid

Adverse drug event (ADE)	LEV	ESM	VPA	STM	LTG	OXC	TPM	Not assignable	Total
Neurologic and psychiatric									
Fatigue	5	1	1	1	1	1		1	11
Disturbance in attention	1		3	1		1			6
Headache	1	2			1		1		5
Mood swings	1	1						1	3
Aggression	2								2
Depressive symptoms	1				1				2
Insomnia	1	1							2
Unsteady gait				1	1				2
Vertigo/dizziness		1		1					2
Agitation	1								1
Mydriasis								1	1
Tremor						1			1
Non-psychiatric/non-neurologic									
Increased appetite/weight gain	1		3		1				5
Vomiting		1		1	1	1		1	5
Nausea		1			1			1	3
Dyspnea				2					2
Decreased appetite/weight loss	1						1		2
Diarrhea							1		1
Exanthema						1			1
Fever							1		1
Liver injury			1						1
Stomach ache		1							1
Total	15	9	8	7	7	5	4	5	60

### Burden of experienced ADEs

Of those children who had experienced ADEs, 32/37 (82%) stated that they were bothered by the experienced ADEs. An emotional burden was described by 11/37 (30%) children, e.g., “It is just disgusting, and nothing is fun anymore,” “You have to take medication and then you get extra problems,” “I was annoyed by it and depending on the situation the side effect made me really angry,” “The aggressions bother me, my mother got loud, there was a row,” and “I woke up every morning and thought: Now I have to face this crisis again.”

Restrictions on school performance due to the experienced ADEs were reported by 8 children, and on favorite leisure activities by 4 children, respectively. Four children were dissatisfied with their own body shape due to an ADE, 4 were bothered that the ADE was noticed by others, and 4 perceived a negative impact on their life due to fatigue. Six children described various aspects bothering them such as nightmares or “Trembling bothers when holding cutlery.”

### Measures taken in case of an experienced ADE

Measures performed in case of an ADE were according to the interviewed children and adolescents (multiple answers possible): consultation of a physician (13/37; 35%), self-management of symptoms (9/37; 24%), change of medication (6/37; 16%), dose reduction (2/37; 5%), and miscellaneous (3/37; 8%), e.g., psychotherapy or alternative medicine. Six of 37 (16%) children stated that no measures were taken and 3/37 (8%) did not know whether any measures had been taken. Of those 8 patients who reported dose reduction or termination of the medication, 6 (75%) stated an improvement of their situation due to the change. Examples for self-management of symptoms were diet for weight control, or caffeine-containing drinks/naps for fatigue.

According to the medical records, in 34/60 (57%) ADEs, the physician did not change the medication; in 10/60 (17%), the anticonvulsant drug was discontinued (in 3 of those patients, the medical records indicated the medication was mainly discontinued because of seizure freedom); in 5/60 (8%), the dosage was reduced; in 1/60 (2%), the co-medication was changed; and in 1/60 (2%), the dosage form was switched. In 3 patients who each described 2 different ADEs under the same monotherapy and 1 patient who described 3 different ADEs under the same monotherapy, it was not possible to assign the physician’s reaction to a specific ADE. According to the medical records, in 2 of those 4 patients describing more than 1 ADE of the same drug the anticonvulsant drug was discontinued (once due to ADEs alone, once due to ADEs and limited effectiveness); in the other 2 patients, the dosage was reduced (once due to ADEs, once due to seizure freedom).

## Discussion

We performed a survey addressing ADEs of anticonvulsant drugs as experienced and described by the pediatric patients themselves. Forty percent of children and adolescents expressed fears that the medication could harm them. About two thirds of those who named specific ADEs mentioned ADEs concerning neurologic or psychiatric symptoms. One third of patients described experienced ADE, again mostly concerning neurologic or psychiatric symptoms. Most patients who experienced ADEs perceived an emotional burden and distinct restrictions in their daily life.

### Fears concerning the medication

It is of concern that 40% of children and adolescents fear that a medication that should first of all improve their health condition and quality of life could harm them or their body. Nevertheless, this fact reveals a highly differentiated view of patients on their medication as anticonvulsants bear a high risk of ADEs. In contrast to parents who mainly expressed fears concerning severe organ damage such as liver failure that seldom occurs [2], children and adolescents mainly feared neurologic and psychiatric ADEs which occur frequently, i.e., very realistic fears. One reason for this might be that we only included participants who were already on anticonvulsant long-term medication. The fears of the children might be shaped more by concrete experiences than by abstract information [12]. Particularly worrying is the fact that three of the interviewed children expressed fears of death and intoxication due to their medication. Of course, this risk exists. However, compared to the risk of a fatal outcome of the epilepsy due to status epilepticus, injuries, or SUDEP (sudden unexpected death in epilepsy) [1, 8, 9], the risk of a fatal ADE is still much lower. In addition to a negative impact on the emotional situation of the child or adolescent, fears of ADEs and death due to medication can have a negative influence on adherence. There are several factors that can influence adherence in pediatric epilepsy therapy [19, 26]. In patients with epilepsy, safety concerns are a common reason for non-adherence to anticonvulsant medication [21]. A poor adherence can worsen the epilepsy [6] with an increased epilepsy-related mortality. Thus, good information on the epilepsy and its risks [15] and on the risk-benefit-ratio of the medication are necessary.

### Experienced ADEs

Almost two thirds of the experienced ADEs concerned neurologic or psychiatric symptoms. Those ADEs are common in anticonvulsant therapy and are also described in the adult population [5, 18, 25]. Those data are in accordance with our parents’ survey [2]. It is of note that even in comparably less severe epilepsy syndromes such as absence epilepsy or rolandic epilepsy (which have a known comorbidity with attention-deficit hyperactivity disorders [17]), a high proportion of patients described experienced ADEs. Sometimes ADEs and symptoms of the disease are difficult to differentiate. If the patient, however, is convinced of having ADEs, this can again have an additional negative impact on adherence. For drugs with a low risk of severe ADEs such as levetiracetam or ethosuximide, the pediatric patients relatively often described less severe but nevertheless burdensome ADEs. Those ADEs could have a high potential of having a negative impact on quality of life as shown in this study and in an earlier parents’ survey [2].

### Burden of experienced ADEs

Epilepsy can have a negative impact on the quality of life of children and adolescents that can be aggravated by a developmental disorder and further aggravated by ADEs [24]. The results of the present study showed a high burden of experienced ADEs with a negative impact especially on the emotional situation, on school performance, and on favorite leisure activities. This is again in accordance with the data of a recent parents’ survey on ADEs of their children’s anticonvulsant medication [2]. All efforts should be made to minimize the negative impact of ADEs on the quality of life of pediatric patients. That is all the more important since children with epilepsy already feel impaired by their disease itself [10]. This negative impact on the patients’ quality of life was also observed by physiotherapists, occupational therapists, and speech therapists [13]. Some children describe a negative impact on their body shape. Especially valproate [7] but also other anticonvulsant drugs such as oxcarbazepine [11] can cause weight gain. Fear of weight gain is especially present in young females [23]. Thus, the risk of non-adherence on a medication that has the potential of causing weight gain is especially high in adolescents, particularly in girls.

### Measures taken in case of an ADE

In some of the children who reported a change in their medication due to an ADE, the situation was not improved. This can be due to several reasons. If the dose of the anticonvulsant was reduced, the reduced dose can still have the potential to cause an ADE. If the medication was changed, either the new medication can have the same ADE or the symptom could have been misinterpreted as ADE but was for example caused by the disease.

The use of alternative medicine in case of an ADE was also reported in this study. In an earlier survey, it was shown that the chance of turning to complementary and alternative medicine was 5.6-fold higher if parents perceived ADE of their child’s anticonvulsant medication [14]. This bears the risk of termination of the necessary routine anticonvulsant medication.

A number of children reported they did not know what was done due to the ADE or that nothing was done. Also, medication was not changed according to the medical records in more than half of the described ADEs by the pediatric patients. This may be acceptable if the ADE is not very severe. Due to the negative impact of ADEs on the overall situation of the children as shown in this study, physicians should address the potential occurrence of ADEs and help pediatric patients and their families either to reduce ADEs or to find options to cope with them.

## Limitations

Children might have difficulties in remembering ADEs that occurred a while ago and in judging symptoms as ADEs. The study, however, focused on the subjective perception of the children and adolescents. Thus, we aimed to explore which symptoms were attributed to a potential ADE by the patients. A cognitive ability to understand and answer our questions was necessary. As many severe epilepsy syndromes often go along with impaired cognitive abilities children with less severe forms of epilepsy were overrepresented. Patients with more severe epilepsy syndromes could have experienced even more ADEs. This fact has to be taken into account when interpreting the data. In addition, the quality of the patients’ medical records was very heterogeneous due to a high number of pretreating physicians and the records did not always contain all necessary pieces of information on ADEs. As the study was performed in two hospitals, the patient collective was limited to 100 participants. Possibly, a broader insight into patients’ perceptions would have been feasible with more patients.

## Conclusion

Children and adolescents are well aware of the ADEs of their anticonvulsant medication. A high percentage of the described ADEs concerns neurologic or psychiatric symptoms. Those ADEs are also described in children and adolescents with comparably less severe epilepsy syndromes such as absences and rolandic epilepsies. Besides, pediatric patients taking anticonvulsant drugs with a low risk of fatal ADEs such as levetiracetam and ethosuximide report less severe but nevertheless burdensome ADEs. According to the patients, those ADEs can lead to an emotional burden and to restrictions especially in favorite leisure activities and school performance. The treating physician and the whole social pediatric team, consisting of physicians, psychologists/psychotherapists, social workers, and therapists, should make every effort to balance seizure control and restrictions of quality of life caused by ADEs of anticonvulsants.

## Abbreviations

ADE(s): Adverse drug event(s)
SUDEP: Sudden unexpected death in epilepsy
